# Supplementary material for: Efficient self-organization of informal public transport networks
Source: Nat Commun. 2024 Jun 8;15:4910. doi: 10.1038/s41467-024-49193-1 (PMC11162447; doi:10.1038/s41467-024-49193-1)
Supplement: Supplementary file 1 — Supplementary Information [file 41467_2024_49193_MOESM1_ESM.pdf]

Supplementary Information

accompanying the manuscript

Efficient self-organization of informal public transport networks

Kush Mohan Mittal,[1] Marc Timme,[1,2] and Malte Schröder[1]

[1] *Chair of Network Dynamics, Institute of Theoretical Physics and Center for Advancing Electronics Dresden (cfaed), TUD Dresden University of Technology, 01062 Dresden, Germany*

[2] *Lakeside Labs, 9020 Klagenfurt, Austria*

In the main manuscript we revealed structural features of informal public transport networks in the Global South compared to their formal counterparts in the Global North. We have introduced three variables to quantify the structure of these networks: detour heterogeneity ξ, total detour D, and intermediate routes C. Our comparison shows that informal transport not only compares to but often also outperforms formal transport. This Supplementary Information provides additional details for the data, methods, and the results presented in the main manuscript.

Data and Methods

Supplementary Note 1 Route Data
Supplementary Note 2 Method Details

Sample City Analysis: Cochabamba

Supplementary Note 3 Self-organization of Routes to Population Distribution
Supplementary Note 4 Robustness of Observable
Supplementary Note 5 Fixed Route Length Scan
Supplementary Note 6 Properties of Individual Routes
Supplementary Note 7 Street Type Correlation With Detour
Supplementary Note 8 Detour Heterogeneity vs. Total Detour
Supplementary Note 9 Spatial Route Model

City Comparison

Supplementary Note 10 Detour Profiles
Supplementary Note 11 Individual Route Structure Comparison
Supplementary Note 12 Route Interconnectivity
Supplementary Note 13 Population Weighted Measures

DATA AND METHODS

In the following Supplementary Notes we provide additional details about the data and methods employed in the main manuscript.

SUPPLEMENTARY NOTE 1: ROUTE DATA

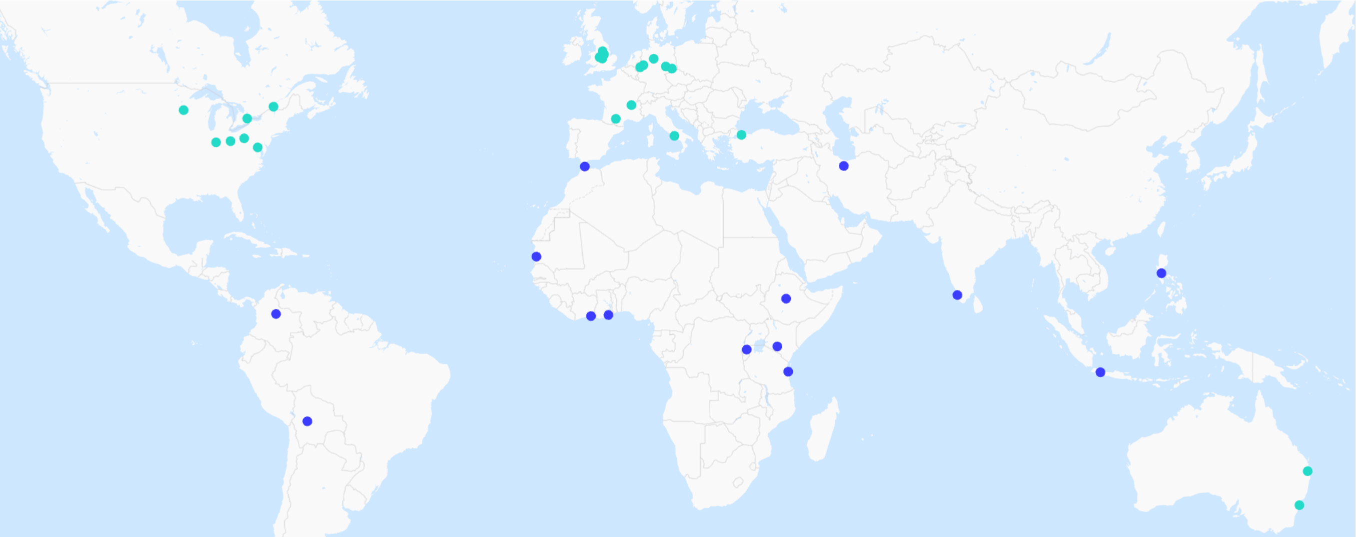

Supplementary Figure S1. Cities (dots) analyzed in the main manuscript distributed across the globe. Cities in the Global South (South America, Africa, South-East Asia) rely on informal transport (dark blue) while the cities with formal transport (light blue) are predominantly in the Global North (North America, Europe, Australia).

In the main manuscript, we analyze bus routes from 36 cities across the globe. The cities cover both formal, centrally organized public transport in developed countries and informal, self-organized public transport in developing countries in the Global South (Fig. S1). Cities in developing and developed countries differ significantly in their topology and structural properties such as area and population distribution. The differences between the structure of formal and informal transport routes observed in the main manuscript may have various reasons. For example, one might expect that routes of informal transport services have less detour and are straighter because of a higher density of routes in these regions, implying less area to be served by one particular route and hence a straighter overall bus route. Here we show that there are no significant statistical differences between the cities with formal and informal transport studied in the manuscript. In particular, Tab. S1 shows data on the number of routes, area, and population served by buses in all 36 cities.

Due to varying definitions of city boundaries and urban areas, we directly compute the relevant area and population of all cities based on the service area of the buses. Using hexagons at a 400m resolution extracted from the Kontur Population Dataset (see Methods in the main manuscript and Fig. S6c for an illustration of the population distribution in Cochabamba), we calculate the area and population of a city as the total area of all hexagons served by at least one bus route and the total population in these hexagons, respectively. The values in Table S1 may therefore differ from official data. This does not affect our structural analysis of the routes but neglects other dimensions of public transport provision such as accessibility of the city in terms of the fraction of the city covered by (bus) public transport.

In both formal and informal public transport, it is common for a particular bus with a given bus ID in a city to have multiple routes associated with it. These routes may differ based on factors such as the direction of travel, for example due to the presence of one-way streets, changes in the routes during different times of the day, or the preferences of drivers in informal transport, who may dynamically tweak their routes to maximize their profit. To account for these variations, we refer to the total number of routes in a city as *bus routes* (Tab. S1 third column), while the number of distinct buses is represented by the number of *unique bus IDs* (Tab. S1 third column in brackets). Informal transport in cities typically has a median of 2.0 routes per ID (1.9 to 2.1 in the 25/75th percentile), whereas formal transport tends to have 1.4 routes per ID (1.0 to 2.4 in the 25/75th percentile). These figures are consistent with the notion

that informal transport routes have a consistently high variability and do not stringently follow prescribed routes. However, these numbers also do depend on the local definition and numbering of routes or bus ID and as such may not be directly comparable across all cities.

The density of bus routes is similar for both formal and informal transport. Specifically, cities with informal transport have a median of $0.59\,\text{routes/km}^2$ with high variation from 0.48 to $1.16\,\text{routes/km}^2$ in the 25/75th percentile. In contrast, cities with formal transport have a median density of $0.43\,\text{routes/km}^2$ with a relatively smaller variation from 0.23 to $0.59\,\text{routes/km}^2$ in the 25/75th percentile. The values suggest that informal transport operates a slightly higher number of routes per unit area. However, it is not necessarily true that the routes in informal transport are straighter due to this higher density, as the standard deviation for both types of transport is high. The variations thus would not explain the consistent difference we find between informal and formal transport in terms of detour heterogeneity and total detour. Furthermore, the apparent difference in the number of routes per unit area is not as significant as it may seem, since in informal transport, similar routes with minor variations are often 'double counted' due to the higher number of routes per bus ID, whereas this is not as prevalent in formal transport.

In contrast to the area-density of the routes, the number of routes per person in the area covered by routes is significantly smaller for informal transport. Cities with informal transport have a median $0.0825, \text{routes}/10^3\text{population}$ (0.0427 to 0.2612 in the 25/75th percentile), cities with formal transport have $0.1327\,\text{routes}/10^3\text{population}$ (0.0863 to 0.2112 in the 25/75th percentile). This mirrors the underlying economic incentives to operate informal transport in high-density areas where the service may be profitable in contrast to subsidized formal public transport.

Overall, these statistics suggest that the structural efficiency and straightness of informal transport routes is not simply a consequence of a higher density of buses or similar systematic differences between cities with formal and informal public transport.

City	Structure	Routes	(Unique ID)	Area / km^2	Population / 10^3	Routes per km^2	Routes per 10^3 Population
Abidjan	Informal	1120	(112)	356	3940	3.14	0.2842
Accra	Informal	564	(282)	354	2933	1.59	0.1923
Addis Ababa	Informal	103	(52)	213	2464	0.48	0.0418
Bandung	Informal	68	(33)	123	2810	0.55	0.0242
Brisbane	Formal	35	(19)	185	384	0.19	0.0909
Cochabamba	Informal	431	(130)	342	1058	1.26	0.4071
Columbus	Formal	44	(41)	532	756	0.08	0.0581
Coventry	Formal	37	(37)	186	436	0.2	0.0847
Dar es Salaam	Informal	323	(151)	365	4401	0.88	0.0734
Dortmund	Formal	215	(73)	268	656	0.8	0.3273
Dresden	Formal	93	(39)	198	507	0.47	0.1831
Duitama	Informal	58	(26)	76	166	0.76	0.3488
Dusseldorf	Formal	223	(82)	395	1068	0.56	0.2088
Hanover	Formal	97	(54)	432	700	0.22	0.1384
Indianapolis	Formal	31	(30)	390	481	0.08	0.0643
Istanbul	Formal	230	(224)	854	13917	0.27	0.0165
Kigali	Informal	82	(41)	128	729	0.64	0.1125
Kochi	Informal	918	(918)	594	1844	1.54	0.4977
Leipzig	Formal	172	(71)	306	564	0.56	0.3045
Lyon	Formal	270	(111)	426	1273	0.63	0.2121
Manila	Informal	87	(45)	181	4854	0.48	0.0179
Minneapolis	Formal	399	(314)	966	1409	0.41	0.2832
Montreal	Formal	83	(81)	174	1127	0.48	0.0736
Nairobi	Informal	201	(105)	424	4426	0.47	0.0454
Naples	Formal	179	(92)	179	1271	1	0.1408
Nottingham	Formal	87	(85)	135	518	0.64	0.1679
Nouakchott	Informal	40	(20)	85	740	0.47	0.054
Pittsburgh	Formal	79	(72)	515	621	0.15	0.1271
Sheffield	Formal	79	(51)	295	760	0.27	0.1038
Sydney	Formal	72	(61)	273	1091	0.26	0.066
Tehran	Informal	50	(41)	312	4539	0.16	0.011
Tetouan	Informal	50	(25)	141	545	0.35	0.0917
Toronto	Formal	293	(246)	667	2883	0.44	0.1016
Toulouse	Formal	234	(88)	387	774	0.6	0.3022
Washington	Formal	224	(218)	974	2292	0.23	0.0977
Wolverhampton	Formal	165	(55)	213	761	0.77	0.2166

Supplementary Table S1. Aggregated data on all cities studied. Column 2 indicates if the city is served by formal (city name in dark blue) or informal (city name in light blue) public transport. Column 3 shows the total number of bus routes (number of distinct bus IDs) in the city. Columns 4 and 5 show the total area of the city covered by routes (see text), and columns 6 and 7 show the normalized number of distinct bus routes per area and population.

SUPPLEMENTARY NOTE 2: METHOD DETAILS

Self-organization of lines and route variations

Recurring transportation needs, such as daily commutes, lead to the (self-)organization of routes into fixed and recurring lines, even in informal transport. These routes generally maintain a consistent operational direction and trajectory, as they are accountable to the users who expect them to follow specific lines. However, these routes also possess a level of adaptability to accommodate changes in the city's infrastructure, such as one-way streets, the emergence of new demand centers, or dynamical adjustments to avoid congestion, all with the idea of maximizing driver profits. Although these variations do exist, they are relatively minor and different routes corresponding to the same bus ID predominantly overlap. Figure S2(a-c) depicts routes from three different informal transport buses in Cochabamba, Nairobi, and Abidjan, respectively. Versions (i-iv) represent different variations in routes of the same bus ID, while (v) shows the overlap of all the different lines a bus may take. Buses predominantly follow a fixed trajectory with similar start and end points.

Formal transport buses may also have variations in the possible routes they take, for instance due to different routes in peak and off-peak hours or the presence of one way streets while going back and forth between both ends of the route. Figure S2d illustrates different routes of a formal bus with the same ID from Dusseldorf, Germany with small variations. Overall, for both informal and formal transport in our dataset, the routes for a given bus ID are largely static objects with a defined structure that we can analyze.

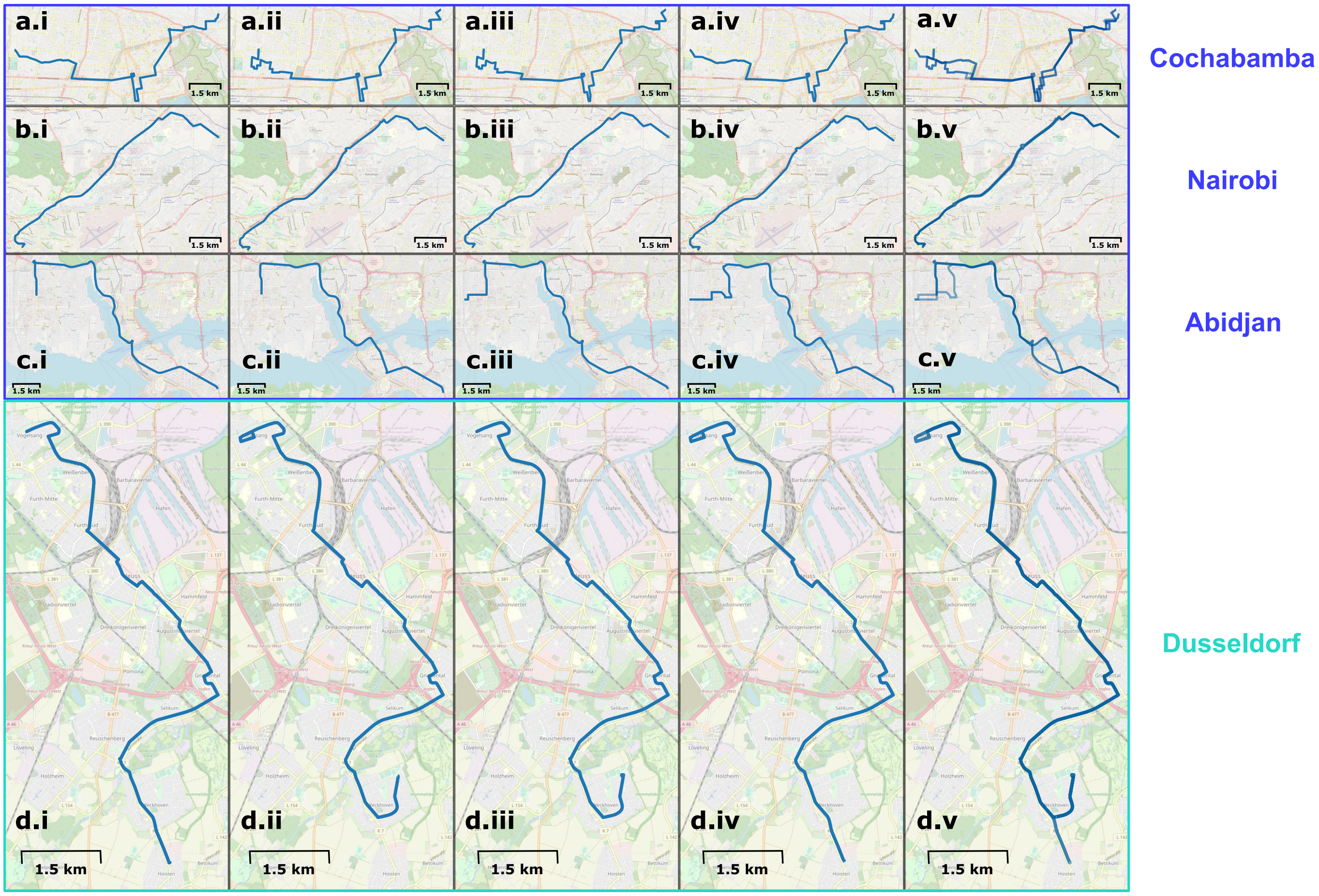

Supplementary Figure S2. High overlap of routes indicates consistent lines. **a-c** Different routes belonging to the same bus ID in informal transport services in the city of Cochabamba, Nairobi, and Abidjan, respectively. The routes may differ because of one way streets, dynamically adjusted stops, or congestion avoidance. Despite these variations, we observe that the differences among the routes are small, and the routes predominantly follow similar trajectories. This consistency makes these routes suitable objects to analyze. **d** A bus with multiple routes is not exclusive to informal transport. Formal transport in Dusseldorf also exhibits multiple similar routes associated with the same bus ID.

We quantify the overlap of the routes by measuring the fraction of the length of two routes of the same bus ID where these routes coincide. For this, we introduce an area with a buffer zone of 30 meters around each route, equivalent to the average distance between consecutive GPS points in our dataset. If the areas of routes intersect, we consider them to coincide (Fig. S3a,b. From this data, we compute the overlap fraction of the routes as the fraction of the combined length of the overlapping parts relative to the average total length of the two routes.

Our analysis reveals that a significant proportion of bus IDs within the dataset are observed in pairs (Fig. S3c, illustrating routes per bus ID in Cochabamba). These paired routes likely represent a trip and return trip of a bus between an origin and destination in opposite directions. The definition of the buffer zone identifies parts of routes to coincide despite potential fluctuations in GPS points during data recording or when the recorded routes differ slightly for buses following the same streets in opposite directions along different lanes.

When computing route overlap for bus IDs with more than two associated routes, we adopt a pairwise approach. We calculate the overlap between all distinct pairs of routes for the given bus ID and then compute the average overlap fraction across these pairs. We ignore bus IDs with only a single recorded route for this analysis. The resulting distribution for all bus IDs in Cochabamba is depicted in Fig. S3d, with a median overlap fraction 0.73 demonstrating consistently high overlap between routes of the same bus ID.

Over all cities with formal transport, the median overlap fraction among routes sharing the same bus ID is 0.9, whereas for all cities with informal transport, it is 0.8. This aligns with the expectation that formal transport buses tend to follow more consistent routes, while informal transport buses exhibit greater route variability. Interestingly, the overlap values are consistently large, even for informal transport. This further supports the above observation that, despite the higher route variation, these routes tend to self-organize and follow mostly consistent lines, making them a viable object for our analysis.

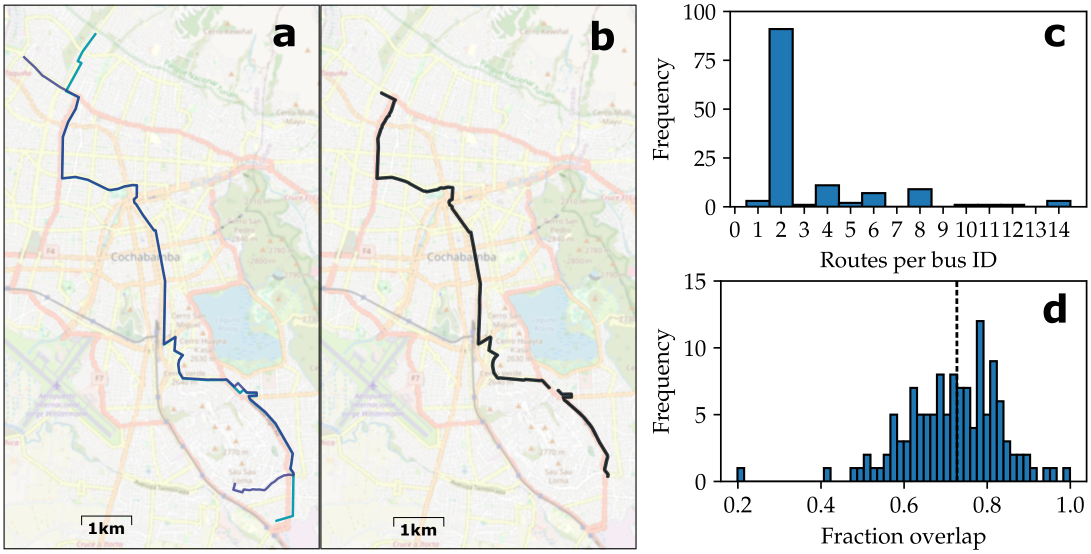

Supplementary Figure S3. Routes with the same bus ID follow similar trajectories. **a** A pair of routes from the city of Cochabamba with the same bus ID. **b** The overlapping part for the pair of routes accounts for 80% of the average total length of the two routes. **c** Histogram for the number of routes per bus ID for the city of Cochabamba. The majority of bus IDs have a pair of routes associated with them, likely representing the same route going in opposite directions. **d** Distribution of the pairwise overlap between routes belonging to the same bus ID for the city of Cochabamba. The median overlap fraction between routes having the same ID in the city of Cochabamba is 0.73 (dashed line). The distribution indicates a consistently large overlap of different routes of the same bus ID.

Computation of the detour profile

An important structural characteristic of the efficiency of a bus route is the detour along the route. We quantify this detour of a bus route by considering shorter segments within the route, each with a length $L/3$, where L represents the total length of the route (see also Methods in the main manuscript). The actual length of each segment may vary due to differences in the distance between successive GPS points. However, this discrepancy is negligible since the spacing of successive GPS coordinates are much smaller than the total length of a route (e.g. 40 m compared to 19 km for the city of Cochabamba, compare also Fig. 2c in the main manuscript).

We parameterize the different segments according to their normalized position $x \in [0, 1]$ along the route. In this context, $x = 0$ signifies the initial segment of the route, spanning a length of $L/3$, and starting from the first GPS point. Conversely, $x = 1$ denotes the final segment of the route, also covering a length of $L/3$, and concluding at the last GPS coordinate of the route. Figure S4a-e illustrates the various segments along a route as x increases.

We analyze the detour on each segment individually by comparing the actual length l_x of the route taken by the bus along the segment at position x to the length s_x of the shortest path between the start and end of the segment. This shortest path is computed with respect to the city road network including, for example, one-way roads. From these lengths, we compute the detour fraction $d_x = \frac{l_x - s_x}{l_x}$ to quantify the normalized detour along each segment x (Fig. S4f). This normalization by l_x allows for comparisons across routes of varying lengths and slightly varying segment lengths along a single route. The detour fraction thus provides a measure of how much the route length could be reduced for a given segment along the route (assuming only the start and end point are relevant stops), effectively describing how much detour the bus takes on different part of the route.

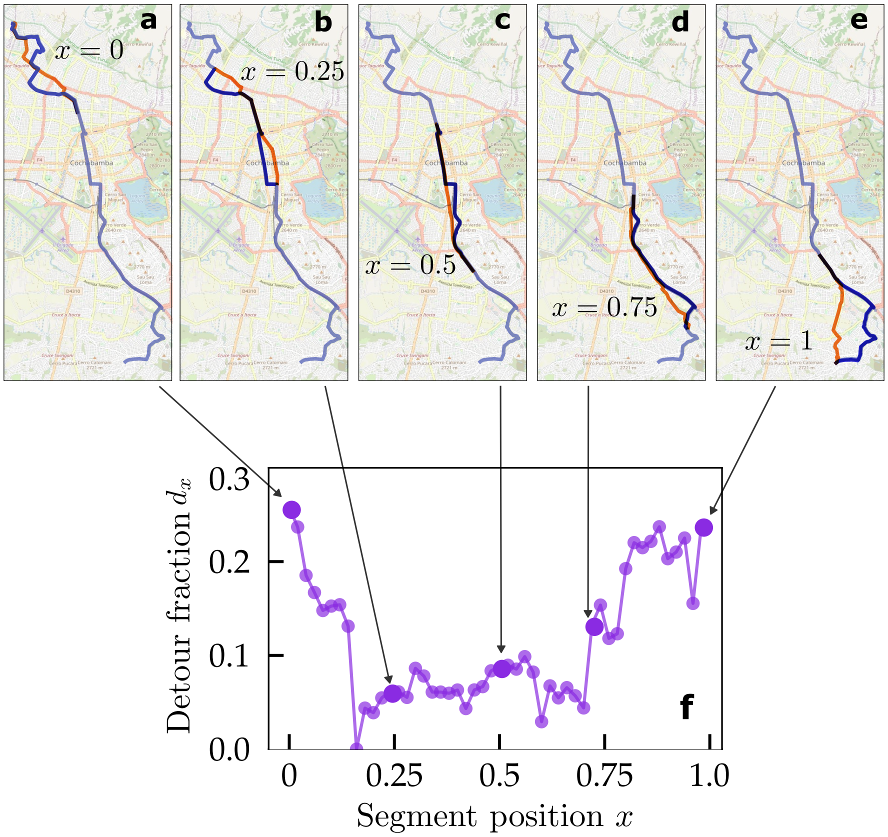

Supplementary Figure S4. Calculating the detour profile of a route. **a-e** Examples of segments (dark blue) of similar length $L/3$ along a route (light blue) of total length L. $x \in [0, 1]$ describes the position of the segment along the route with $x = 0$ denoting the first possible and $x = 1$ denoting the last possible segment. To calculate the detour for a specific segment of a bus route, we compare the length l_x of actual path taken by the bus (dark blue) to the length s_x of the shortest path (orange) between the start and end of the segment. **f** The detour profile of a route describes the relative detour $d_x = (l_x - s_x)/l_x$ across all segments $x \in [0, 1]$ along the route, illustrated here for 50 segments of length $L/3$. Segments shown in panels **a-e** are highlighted by large dots (black arrows).

Interpreting the detour profile

The bus routes observed across the globe exhibit diverse shapes and structures. Their structure is quantitatively reflected in their detour profile. The different shapes of the detour profiles are quantified by two features: the detour heterogeneity $\xi = \frac{d_0+d_1}{2} - d_{0.5}$ (Eq. 3 in the main manuscript) and total detour $D = d_0 + d_{0.5} + d_1$ (Eq. 4 in the main manuscript). Detour heterogeneity ξ measures the fairness of the detour distribution along the route, indicating if the beginning and end of the route experience more detour than the center, $\xi > 0$, or the other way around, $\xi < 0$. The total detour D quantifies the overall inefficiency of the route.

Figure S5 illustrates the relationship between detour characteristics and route structures. A higher value of ξ implies a heterogeneous route with significantly higher detour towards the ends of the route. However, a high value of ξ does not necessarily imply a high overall detour. A route with high ξ may still have a relative small total detour D and be relatively straighter (up to some limit, see Supplementary Note 8) if all the detour is concentrated at the ends of the route. A homogeneous route is characterized by a uniform distribution of detour throughout its length and a low value of ξ. A route with a low value for both ξ and D is predominantly straight and is efficient across the whole route, whereas a high value of D in combination with a low ξ implies a highly inefficient route with high detours throughout its length. A route may also be structured in such a way that it exhibits a negative value for ξ, indicating a relatively higher detour in the middle of the route compared to the ends. The overall detour for such routes is again determined by the value of total detour D.

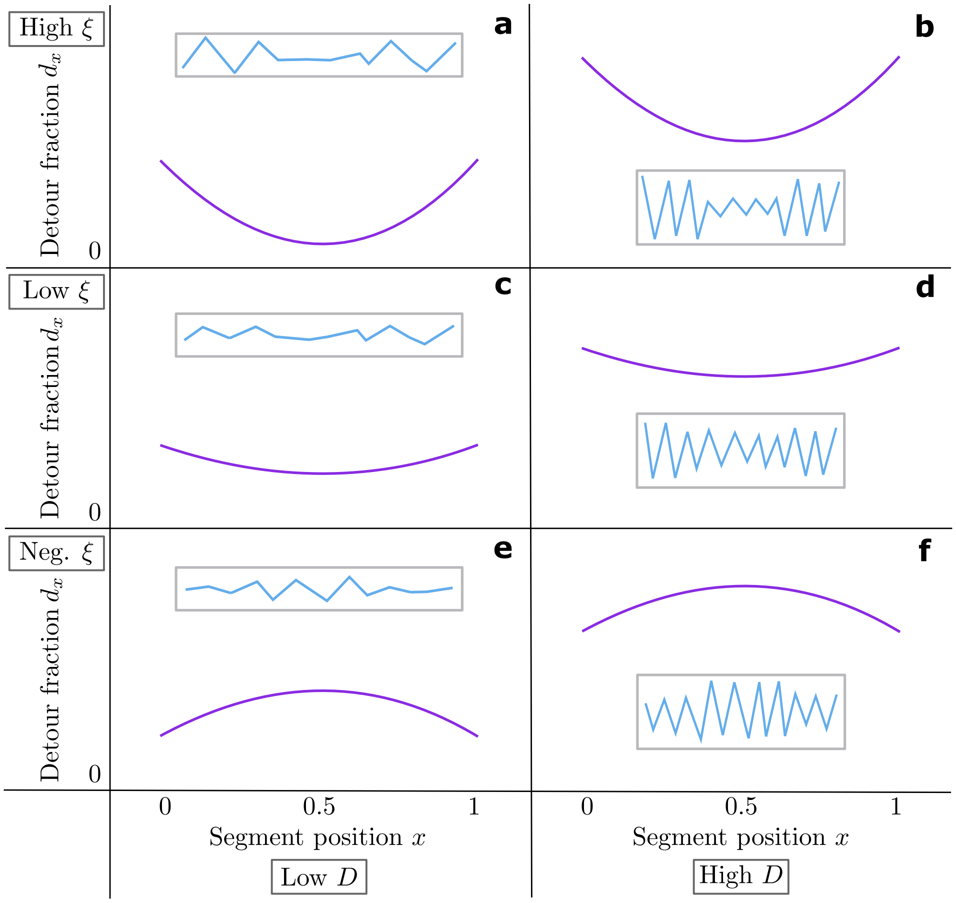

Supplementary Figure S5. Different route structures result in distinct detour profiles. Two metrics summarize the shape of the detour profile (purple) of a route (boxed sketch): detour heterogeneity ξ, which assesses the additional detour towards the route ends, and total detour D, which measures the overall detour along the route. **a,b** A route characterized by a high detour heterogeneity ξ exhibits concentrated detour towards the route ends (u-shaped detour profile). The total detour D determines the extent of the detours (vertical shift of the detour profile). **c,d** A low value of ξ results in a uniform distribution of detour across the route (flat detour profile). A route is structurally efficient when it exhibits both low ξ and low overall detour D (panel c). **e,f** Routes may also have higher detour in the middle of the route compared to the ends, implying $\xi < 0$ (inverse u-shape).

SAMPLE CITY ANALYSIS: COCHABAMBA

In the following Supplementary Notes we provide additional details about the routes from the city of Cochabamba analyzed as an (atypical) example of informal transport routes in the main manuscript.

SUPPLEMENTARY NOTE 3: SELF-ORGANIZATION OF ROUTES TO POPULATION DISTRIBUTION

As illustrated in the main manuscript, the routes of informal transport are not random but rather self-organize efficiently to have low detours. The emerging self-organization of the routes (compare also Supplementary Note 1 and 2) is also evident from the number of routes passing through a region and the population of that region: Fig. S6 shows the relation of the number of routes per population in each of the 400m-hexagons from the Kontur Population Dataset in Cochabamba (compare Methods in the main manuscript and Supplementary Note 1, see also Fig. 1 in the main manuscript). While there are substantial variations, we find that on average the number of routes passing through a hexagon increases with the population in the hexagon. This observation implies that the informal transport routes are not random in their structure and distribution across the city but self-organized to serve the population efficiently.

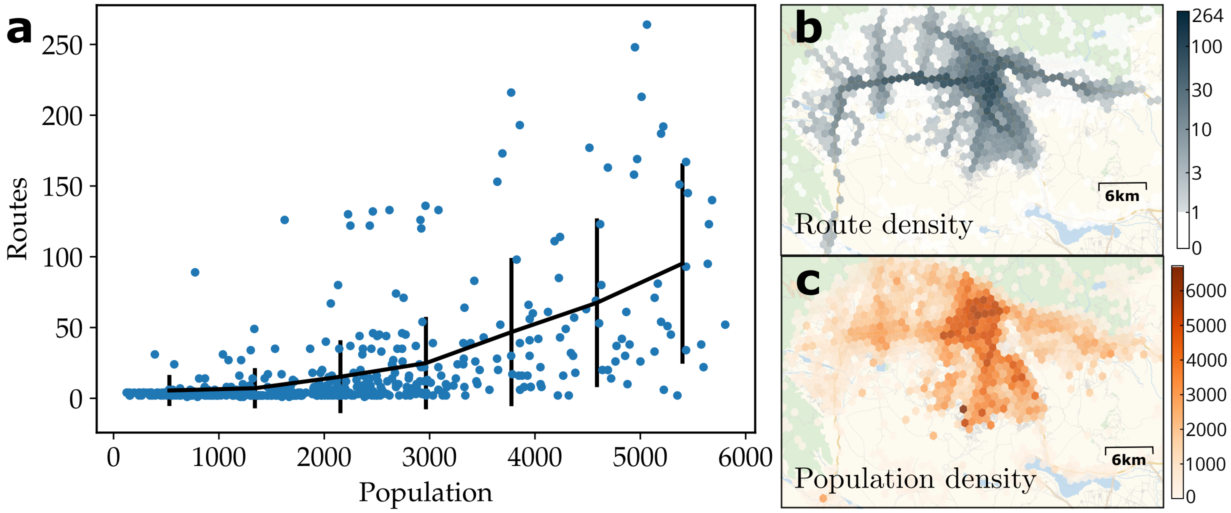

Supplementary Figure S6. Informal transport routes in Cochabamba self-organize with respect to the population distribution. **a** The number of routes passing through an area increases with the population of the area. Blue dots show individual hexagonal areas (compare panel b,c), the black lines show binned average and standard deviation. **b,c** Number of routes and population per hexagonal area (from the Kontur Population Dataset, compare Methods in the main manuscript and Supplementary Note 1).

SUPPLEMENTARY NOTE 4: ROBUSTNESS OF OBSERVABLE

The majority of routes in Cochabamba have a high detour heterogeneity and longer detour towards either end. However, the structure of routes in Cochabamba varies significantly. Fig. S7 shows the detour profile of three individual (first three rows) from the city of Cochabamba. These routes are (i) an example of a route with an atypical structure (i.e. negative ξ) where the middles segment has higher detour compared to the ends (Fig. S7a-d), (ii) a typical route that fits the schematic structure illustrated in Fig. S9d with almost no detour in the middle (Fig. S7e-h) and (iii) a typical route similar to the example given in the main manuscript (Fig. S7i-l). Overall, the typical (median) route structure is similar to the last example such that the detours are much higher towards the ends of a route compared to the middle (Fig. S7m-p).

In addition to these examples, we also test the robustness of our route structure quantification. In our analysis of the route structure and to compute the detour profile, we consider partial segments of the routes with a fixed length $L/3$ where L is the total length of the route (corresponding to segments approximately 6.5 km long for routes with an average length of 19.5 km in Cochabamba). This choice of segment length exactly divides route into middle and end parts, aiding in the interpretability of the detour heterogeneity ξ and the total detour D. Our results remain qualitatively unchanged for different choices of the segment length (different columns in Fig. S7). Choosing longer segments ($L/2$, corresponding to segments with length 9.8 km, Fig. S7d,h,l,p), the segments partially overlap and the differences between the segments become slightly smaller. For shorter segments ($L/4$ or $L/5$, corresponding to segments with length 4.9 km and 3.9 km, respectively), the differences between the segments and the relative detours per segment become larger. This effect is most visible in the median detour profile (Fig. S7m-p). For segment length $L/5$, for instance, the median detour fraction of the middle segment is almost zero while the detour fraction remains high towards the end. For even shorter segments, the discrete local structure of the street network begins to affect the results more strongly. Intriguingly, for larger segments lengths, a slight asymmetry emerges in the median detour profile, suggesting that detours are slightly larger towards the end of a route compared to the beginning. However, this effect is small compared to the detour heterogeneity and total detour of the routes. Overall, this analysis reveals that the majority routes are almost exclusively straight for a substantial length in the middle of route and only take detours towards the very ends of the routes.

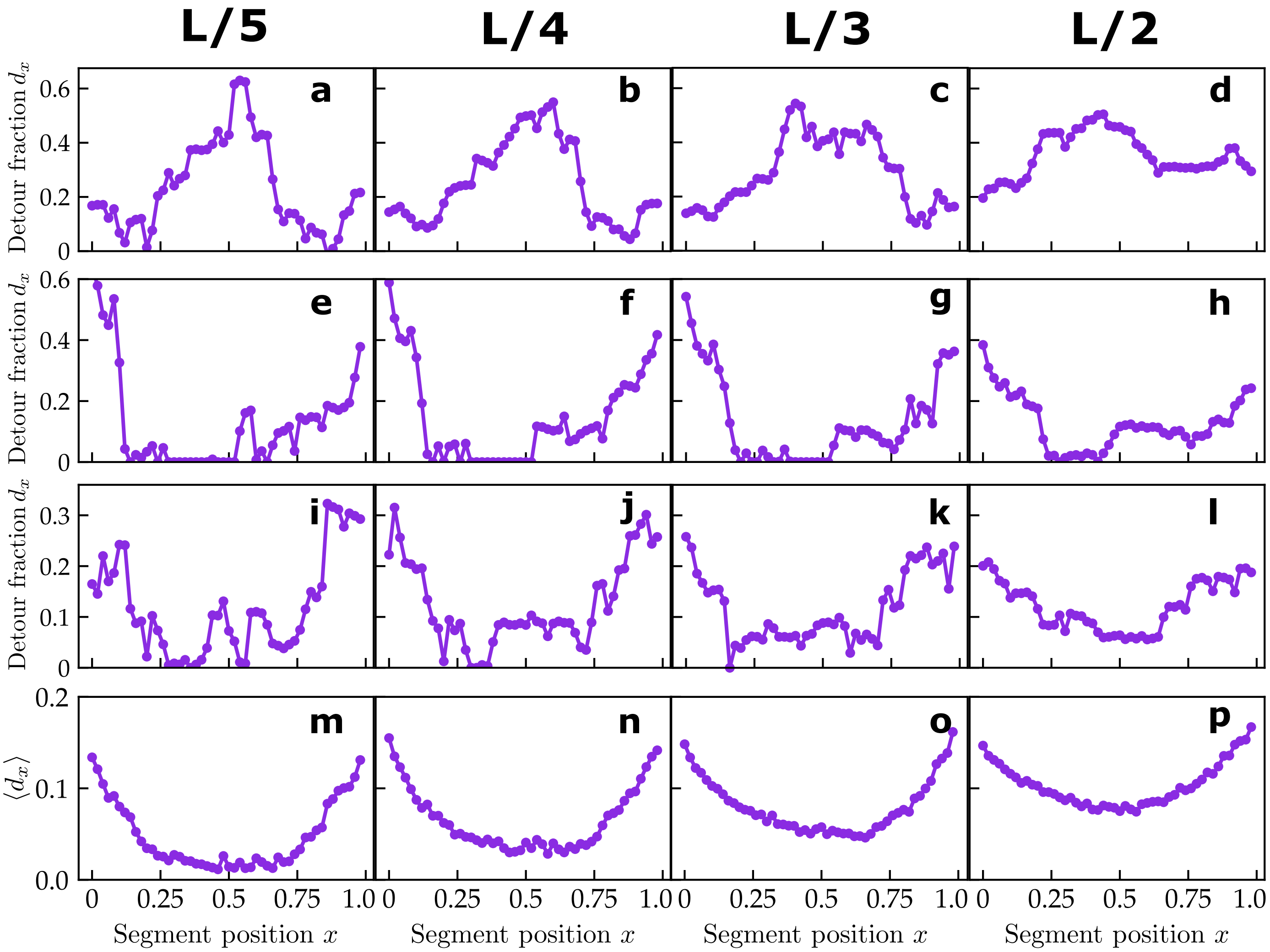

Supplementary Figure S7. Detour profile of three routes in Cochabamba for different segment lengths. **a-d** Only few routes in Cochabamba have a structure with higher detour in the middle relative to the end. **e-h** Many routes have almost no detour in the middle of the route. **i-l** Most routes lie in between both extremes and have high detours towards the ends compared to relatively small detours in the middle. The length of the segments ($L/5$ to $L/2$, different columns) considered for calculating the detour profile does not affect the detour fraction qualitatively (compare along a row for each route). **m-p** The qualitative shape of the median detour profile is similarly robust for different choices of the segment length. The median detour fraction of routes is always higher towards the ends of the routes than middle.

SUPPLEMENTARY NOTE 5: FIXED ROUTE LENGTH SCAN

In the main manuscript, the analysis of detours relies on segments measuring $L/3$ in length, where L represents the total route length. In Supplementary Note 4 (see above), we further demonstrate the robustness of detour distribution results when considering segments of various length fractions. Here, we explore the behavior of detour distributions for routes by utilizing fixed-length segments ranging from 3 to 6 kilometers (see Fig. S8). We find detour distributions similar to those examined in our robustness analysis in Supplementary Note 4 (for the same routes and the aggregated data).

Our primary findings remain robust: detours are concentrated at the ends of the route regardless of the segment length under consideration. However, while the results maintain their robustness across segments of varying fixed lengths, analyzing the observed variables and making comparisons across routes of different lengths becomes difficult when considering segments of fixed length. Considering segment length of $L/3$ allows for the route to be divided into three equal-length segments, leading to an intuitive definition of observables like ξ and D, and enabling comparisons across routes of differing lengths.

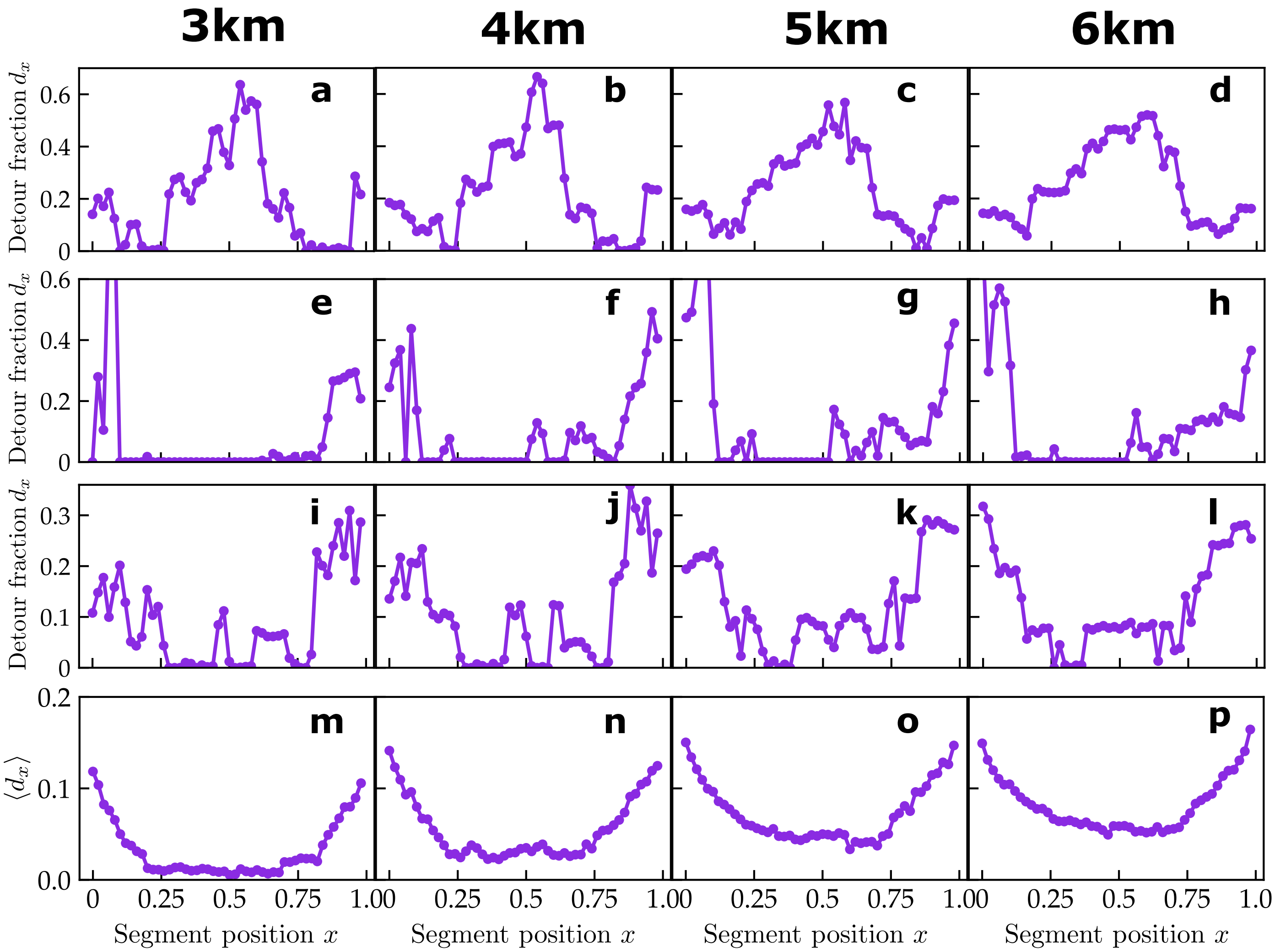

Supplementary Figure S8. Detour profile of three routes in Cochabamba (same as in Fig. S7) for different fixed segment lengths of 3 to 6km. **a-d** Only few routes in Cochabamba have a structure with higher detour in the middle relative to the end. **e-h** Many routes have almost no detour in the middle of the route. **i-l** Most routes lie in between both extremes and have high detours towards the ends compared to relatively small detours in the middle. The length of the segments ($3km$ to $6km$, different columns) considered for calculating the detour profile does not affect the detour fraction qualitatively (compare along a row for each route). **m-p** The qualitative shape of the median detour profile is similarly robust for different choices of the segment length. The median detour fraction of routes is always higher towards the ends of the routes than middle. The results are qualitatively the same as those shown in Fig. S7 in Supplementary Note 4 above, illustrating the robustness of our results regardless of the specific choice of the segment lengths.

SUPPLEMENTARY NOTE 6: PROPERTIES OF INDIVIDUAL ROUTES

Most routes in Cochabamba exhibit a qualitatively similar heterogeneous detour profile, resulting in a positive detour heterogeneity $\xi = (d_0 - 2\,d_{0.5} + d_1)\,/2$ (Eq. 3 in the main manuscript, see also Supplementary Note 2), and a large total detour $D = d_0 + d_{0.5} + d_1$ (Eq. 4 in the main manuscript). However, the quantitative detour profiles d along a route vary substantially between different routes, for example due to routes serving different parts of the city with different street network structure, routes serving smaller areas, or differences in driver behavior and preferences when choosing their routes. While numerous factors impact the detour heterogeneity of routes, certain city parameters, including the route length, fraction of route length in the city center, and population served by the route per unit length, exhibit weak correlations with the detour heterogeneity ξ (Fig. S9).

Detours along longer routes are on average more heterogeneous than for shorter routes (Fig. S9a). This pattern could be due to the fact that longer routes typically serve the outskirts of the city, with the primary objective of transporting people from the outskirts to the city center and vice versa rather than transporting people to different destinations within the city center. As a result, they may pass straight through the city center, stopping only at a few major hubs in the middle of their route.

As a simple test of this hypothesis, we define a circular central region, where the population density is highest, as the city center (black circle in Fig. S9d) and compute the detour heterogeneity of routes in and passing through this region. Routes that primarily serve areas outside the city center (with a small fraction of their length in the city center) tend to have higher detour heterogeneity ξ values than routes operating within the city center (Fig. S9b), consistent with the previous argument.

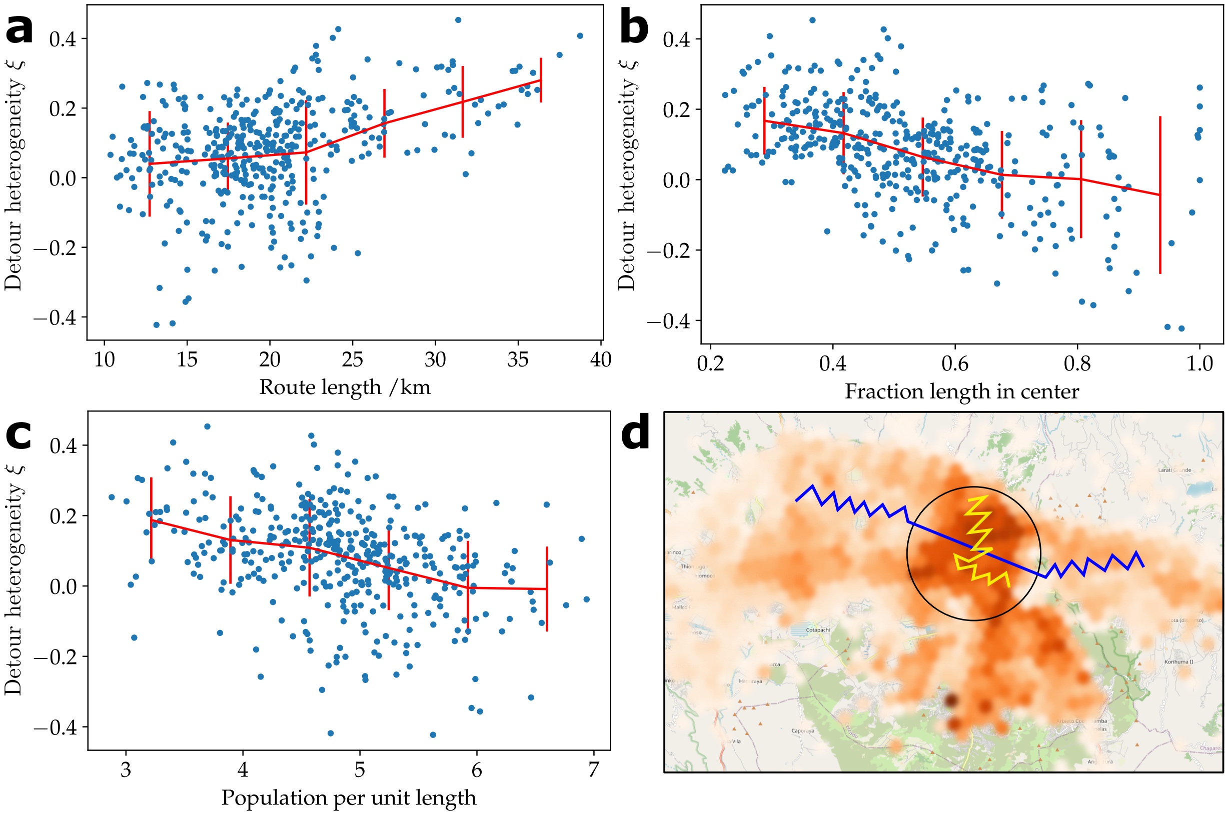

Supplementary Figure S9. Detour heterogeneity ξ of individual routes in Cochabamba is weakly correlated with different route parameters. Each data point represents a single route. **a** On average, longer routes have higher values for detour heterogeneity ξ. **b**, **c** Routes that cover predominantly the city center (circle in panel **d**) and routes that serve more passengers per unit length (e.g. in high/density areas like the city center) have lower detour heterogeneity ξ on average. **d** Schematic illustration of the corresponding route structure in the city of Cochabamba for a long route spanning the whole city serving a relatively small number of customers per unit length (blue) and a shorter route in the high-population city center (yellow, in the circle).

This pattern is confirmed by the decreasing detour heterogeneity of routes that serve a larger number of customers per unit length (Fig. S9c). Interestingly, this is not because the routes in the city center are straighter overall. Indeed, the total detour shows no clear correlation with the route parameters (Fig. S10) and routes in the city center serving more potential customers per unit length of the route even tend to have a higher overall detour everywhere across the route (Fig. S10b). This suggests two qualitatively different types of routes, schematically illustrated in Fig. S9d, with routes in Cochabamba spanning the whole range between these extreme examples.

All correlations detailed above are consistent, confirmed by the correlation between the different route parameters with each other (Fig. S11). For example, we find that the longer routes are the ones that serve outskirts predominantly and the fraction of length in center decreases with increasing in route length (Fig. S11a). Similarly, as the population density is higher in city center, longer routes serving outskirts are also the routes with less population served per unit length (Fig. S11b). Consequently, routes with a large population served per unit length are predominantly serving the city center (Fig. S11c).

Overall, these results show that the detour heterogeneity ξ of individual routes reflects the diverse structure of the city and the different types of routes in the city. The routes with high detour heterogeneity ξ are generally longer, serve city outskirts and have therefore less population served per unit route length as shown in Fig. S9d.

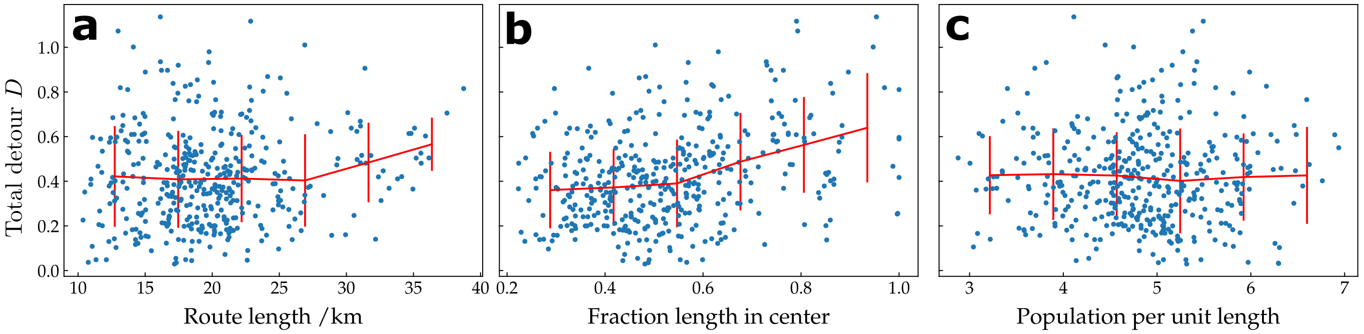

Supplementary Figure S10. Total detour D of individual routes in Cochabamba is largely uncorrelated with different route parameters. Each data point represents a single route. **a,c** The length of a route or the population served per unit length of the route do not significantly affect the total detour D. **b** In contrast to the detour heterogeneity (Fig. S9b), the average total detour slightly increases for routes in the city center.

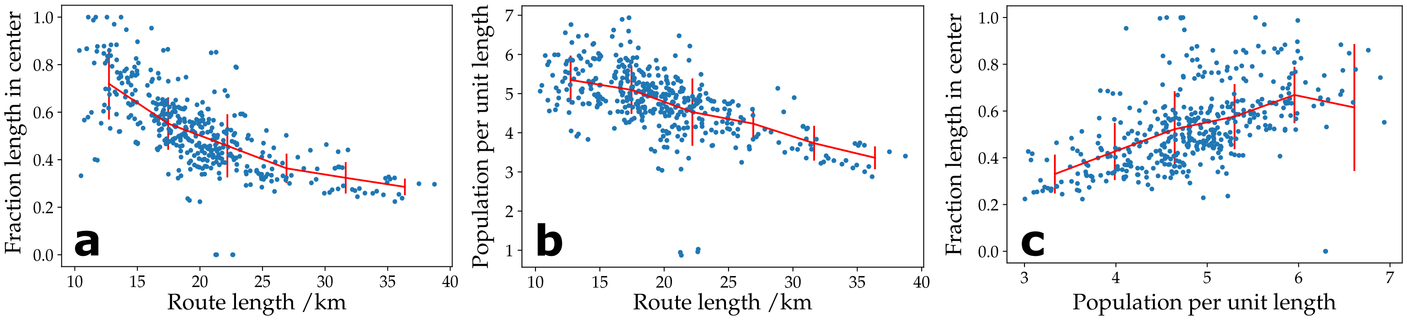

Supplementary Figure S11. Consistent relations between parameters of the routes. **a** Longer routes are also the routes serving the outskirts and have less fraction of length in center. **b** Since the longer routes serve regions in the outskirts with smaller population density, the population served per unit length also decreases with increasing route length. **c** Consistent with previous observations, the population per unit length increases with an increasing fraction of length in city centre.

SUPPLEMENTARY NOTE 7: STREET TYPE CORRELATION WITH DETOUR

Streets within a city can be classified into different categories depending on their purpose, width, and usage (OpenStreetMap, see Ref. [54] in the main manuscript). Here we analyze the correlation of the street type taken by a route segment with the detour along the segment to check if detours are more likely along different types of streets. We take segments of $L/3$ as in the main manuscript and compare the fraction of the length traveled on a specific street type with the detour fraction of that segment. To avoid data quality issues with the street types entered in OpenStreetMap, we consider only major street types Trunk, Primary, Secondary, and Residential as used in OpenStreetMap. All streets without any of these classifications are summarized in the category 'Others'.

Detours are almost evenly distributed across the street types. Detours occur substantially less for routes along highways (trunk), likely due to these segments of a route covering large distances with fewer intermediate stops (compare Tab. S2, last column). In contrast, detours occur slightly more often along trips through smaller streets (secondary). Detours along residential streets do not seem more likely than expected, probably due to the abundance of this street type, thus averaging out potential effects along individual routes. The results are consistent with our previous analysis of the location of detours along a route within the city center or on the outskirts of the city (Supplementary Note 6).

Street type	Segment fraction	Detour fraction	Detour fraction/ Segment fraction
Trunk	0.116	0.053	0.457
Primary	0.137	0.149	1.088
Secondary	0.121	0.137	1.132
Residential	0.465	0.498	1.071
Others	0.163	0.162	0.994

Supplementary Table S2. Comparison of the length of route segments on each street type with the corresponding detour of the route segment averaged over all route segments in Cochabamba. Column 1 denotes the major type of street segments that a bus route passes through as classified by OpenStreetMap. Here 'Others' denotes the sum of the remaining streets not falling in any of the previous major categories. None of the individual street classifications in this group contributes a substantial fraction of the bus route length. Column 2 denotes the average fraction of the segment length travelled on the different street types, computed as the average over all routes and over all 50 positions of segments of length $L/3$ for each route. Column 3 denotes the corresponding normalized detour fraction attributed to each street type, e.g., counting half the detour fraction of the route segment to a street type if half of the length of the segment travelled along streets of that type. Overall, segments along trunk roads (e.g. highways) contribute substantially less detour than expected (last column), likely due to these segments representing long stretches of a trip along a short path between distant parts of the city without stops in between. There is no discernible influence of the other street types, all roughly corresponding to the expected amount of detour.

SUPPLEMENTARY NOTE 8: DETOUR HETEROGENEITY VS. TOTAL DETOUR

The analysis above indicates that total detour D and detour heterogeneity ξ vary strongly across different routes. As described in the Methods section in the main manuscript, these parameters are not independent of each other. They are related mathematical constraint for the detour heterogeneity $\xi \leq D/2$ for a given D. This boundary is attained when the detour fraction in the middle segment of a route is zero ($d_{0.5} = 0$), and the entire detour is focused on the end segments (d_0, d_1). A similar constraint also applies in the case of routes where the detour is centered in the middle segment, with the boundary $\xi \geq -D$ being relevant.

Comparing the total detour D and the detour heterogeneity of the individual routes shows that most routes are at or close to the maximum possible heterogeneity (Fig. S12a). Only routes serving predominantly the city center lie further away from these theoretical limits and exhibit a high total detour compared to a relatively small detour heterogeneity (Fig. S12b). This observation further supports the schematic structure of the routes illustrated in Fig. S9d.

We note that all of the above analysis is particular for Cochabamba. Random sampling of other cities show that for most cities similar correlations do not exist to the same degree everywhere.

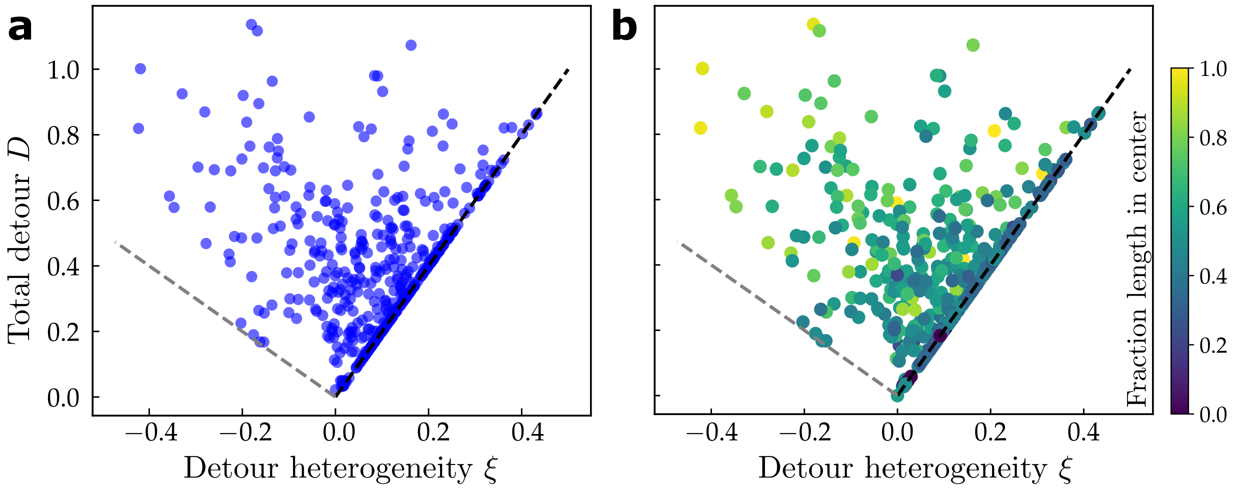

Supplementary Figure S12. Constraints on the total detour D and detour heterogeneity ξ for individual routes in Cochabamba. **a** Along most routes, the detour is distributed as heterogeneously as possible (high ξ, dashed black line) with the detour concentrated in the beginning and end of the route while the middle segment of the route is straight. **b** Only routes that operate mostly in the city center deviate from this limit and exhibit a relatively high total detour with a low detour heterogeneity.

SUPPLEMENTARY NOTE 9: SPATIAL ROUTE MODEL

One natural question that arises is how far the observed detour heterogeneity ξ is caused by the spatial structure of a city consisting of city centre and outskirts. In a typical route within a city, buses start and end in the outskirts and pass through the city center. Already a simple geometrical argument suggests that the area an individual bus covers is smaller in the center and larger in the outskirts. A simple visualization of this idea are two circular segments for the area served by a bus with opening angle θ. To serve the entire area with a spacing of at most $2r$ between different parts of the route would require a route as illustrated in Fig. S13a,b. In this highly simplified representation, the distance r represents the maximum distance people would walk to the bus. The opening angle θ is determined by the total number of buses covering the whole city. Finally, the total length L of the route captures the spatial extent of the city.

Computing the detour profile for the model route depicted in Fig. S13a similar to the analysis carried for the bus routes in cities reveals a qualitatively similar profile as observed in the main manuscript (Fig. S13c). Here, we again take segments of length $L/3$ as in the main manuscript and take the shortest distance as the euclidean distance between the points here. Adjusting the models parameters to approximately represent the conditions in Cochabamba, we set the total route length as $L = 20$km (similar to the average length 19.5 km, compare Supplementary Note 4). An opening angle $\theta = 0.5$ degree approximately represents a uniform distribution of the 431 bus routes in Cochabamba (compare Tab. S1) across the full 360-degree circle, since each route covers an angle of 2θ, representing the left and right sections of the circular segments in Fig S13a. Finally, we set the walking distance to small but realistic value of $r = 200$m.

As expected from the model, detours increase towards the ends of the route and are smallest towards the middle. Surprisingly, the detour distribution for the model is also quantitatively remarkably similar to the actual detour distribution for the city of Cochabamba. However, this is likely coincidental as the model strongly oversimplifies the qualitative structure of routes in a city and neglects variations in population density or geography.

Further investigation reveals the impact of changes in model parameters on the detour distribution. Increasing the route length L results in a greater number of arcs along the route. This leads to an overall increase in detour and a further amplification of the detour difference between the ends of the route compared to the middle (Fig. S13d). As the walking distance r increases, the number of arcs a route makes for a given length decreases, decreasing the overall detour for the route segments (Fig. S13e). Finally, the opening angle θ governs the area a route needs to cover. An increase in θ leads to longer back-and-forth arcs along the route and increases the detour along the route (Fig. S13f), similarly to an increase in L. Overall, we find that the qualitative implications of the model are robust with respect to change in parameters value. The observed structure of the detour profile of a typical route may thus partially be explained by a simple city-center-focused organization of the bus routes, automatically resulting in heterogeneous detours.

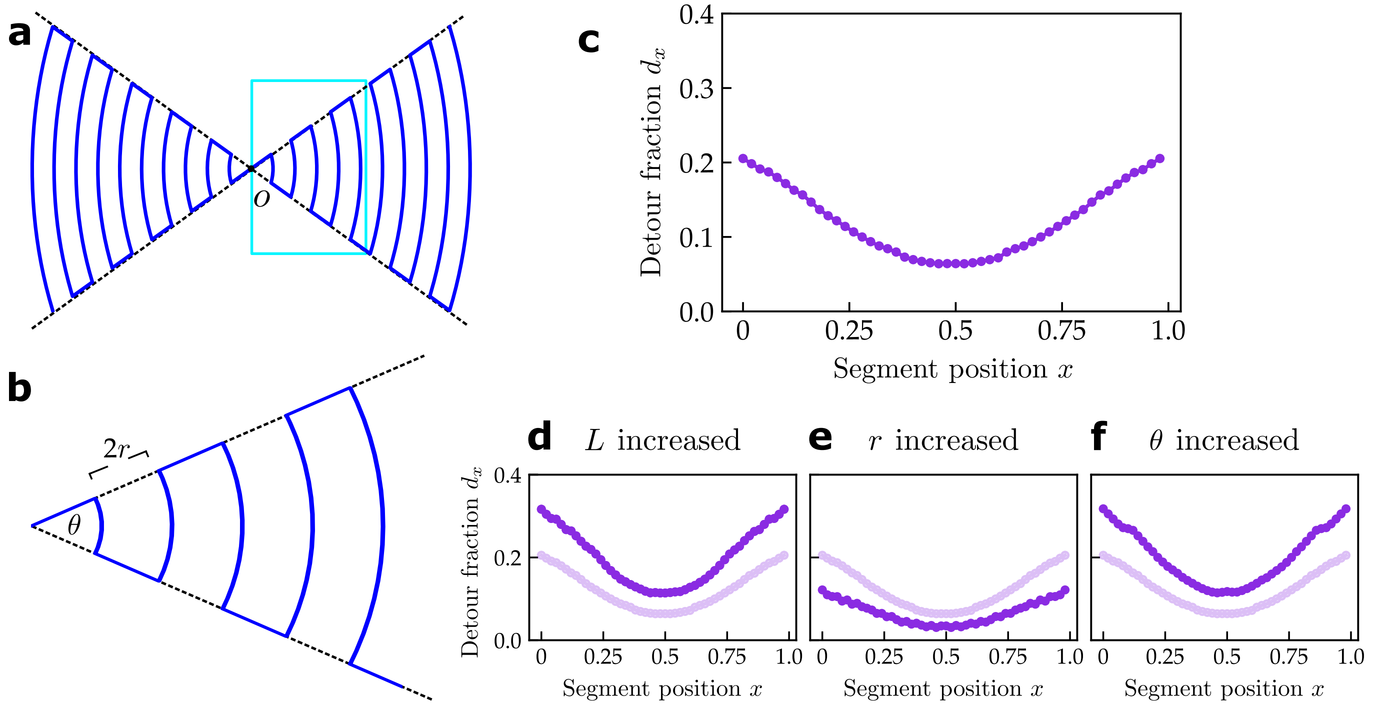

Supplementary Figure S13. Spatial model explains route heterogeneity. The heterogeneity of detours along a route and the qualitative detour profile may be explained by a simple geometric argument. **a,b** Schematic illustration of a bus route covering two circular segments around a city center O with opening angle θ. As the distance to the center increases, longer detours are required to cover the whole area. The parameter r denotes the maximum walking distance to reach the bus line. **c** Detour profile for the model route for $\theta = 0.5\,$degree, $r = 200$m, and a total route length of $L = 20$km. The detour profile is qualitatively similar to the empirical detour profiles observed in the main manuscript. **d-f** The results of the model are qualitatively robust to changes in the model parameters. Detours and detour heterogeneity increase compared to the base case (light purple, compare panel c) for increasing route length (larger distance from the city center, panel d) and a larger opening angle θ (panel f), both resulting in more back-and-forth routes. On the other hand, as the walking distance r increases fewer back-and-forth arcs are required to cover the area and the detour and detour heterogeneity decreases (panel e). Here, all values were individually double compared to panel c.

CITY COMPARISON

In the following Supplementary Notes we provide additional details on the comparison of public transport route structures across different cities as illustrated in Fig. 3 in the main manuscript.

SUPPLEMENTARY NOTE 10: DETOUR PROFILES

The main manuscript reveals that formal transport routes on average tend to have higher detour heterogeneity ξ and total detour D compared to informal transport. This trend is also evident from the median detour profiles of cities falling into each category, as shown in Fig. S14. Specifically, the detour profiles of cities with formal transport (Fig. S14a-h) mostly exhibit higher relative detours especially towards the ends of the routes, indicating a structural inefficiency when compared to overall very low detours in most cities with informal transport (Fig. S14i-p).

Leipzig and Duitama (Fig. S14d and i, respectively) are unique among the analyzed cities. Their public transport routes seem to be structured qualitatively differently compared to other cities and exhibit a higher detour in the middle of the routes and less detour towards the end. While such differences may be the result of the local structure of the street network, for example by limiting the available paths through the city center, the effect is comparatively small and may simply be a result of randomness in the structural properties of the routes (see also Supplementary Note 11).

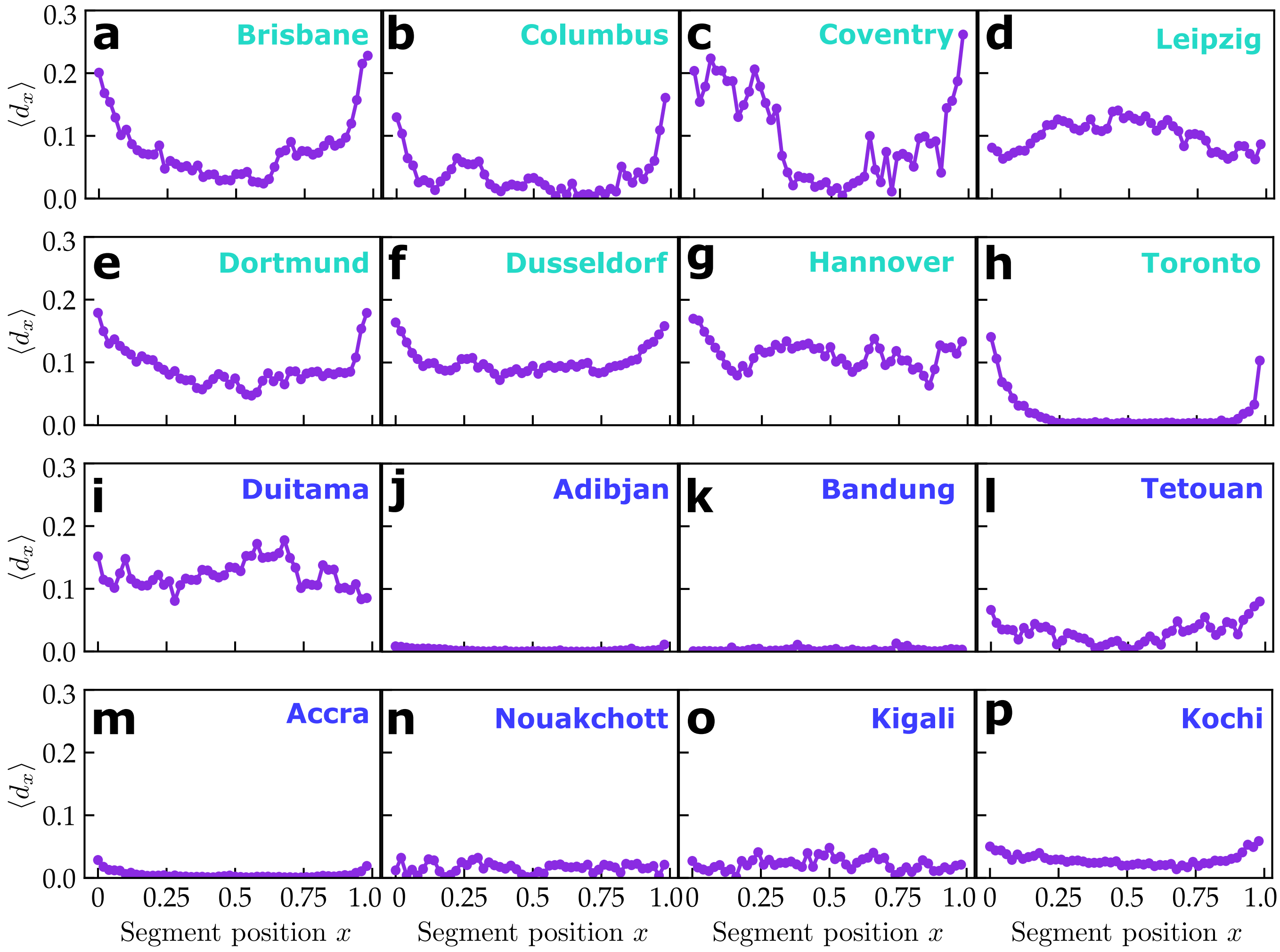

Supplementary Figure S14. Median detour profiles for different cities. Cities with formal public transport (light blue, panels a-h) typically have a detour profile with overall higher relative detour and higher detours towards the ends of the route relative to cities with informal public transport (dark blue, panels i-p).

SUPPLEMENTARY NOTE 11: INDIVIDUAL ROUTE STRUCTURE COMPARISON

To better understand differences between the cities, we repeat the analysis of the detour heterogeneity ξ and the total detour D for the individual routes in the cities (Fig. S15, compare Supplementary Note 8 and Fig. S12). Across all cities, most routes accumulate close the high detour heterogeneity boundary $D = 2\xi$ (see Supplementary Note 5 and Methods in main manuscript). This effect is most visible for cities with formal transport and routes with high total detour Fig. S15a-h. On the other hand, most cities with informal transport routes exhibit smaller values for both D and ξ, Fig.S15(i-p). Again, Leipzig and Duitama are exceptions with the majority of their routes structured with negative detour heterogeneity ξ, though still many routes fall on the high-heterogeneity boundary while almost no routes exhibit the other extreme.

Overall, the analysis qualitatively shows a large similarity between all cities independent of formal or informal transport and illustrates the large variation in route structures within each city. However, quantitatively routes of formal transport have significantly higher values of total detour and detour heterogeneity than informal transport.

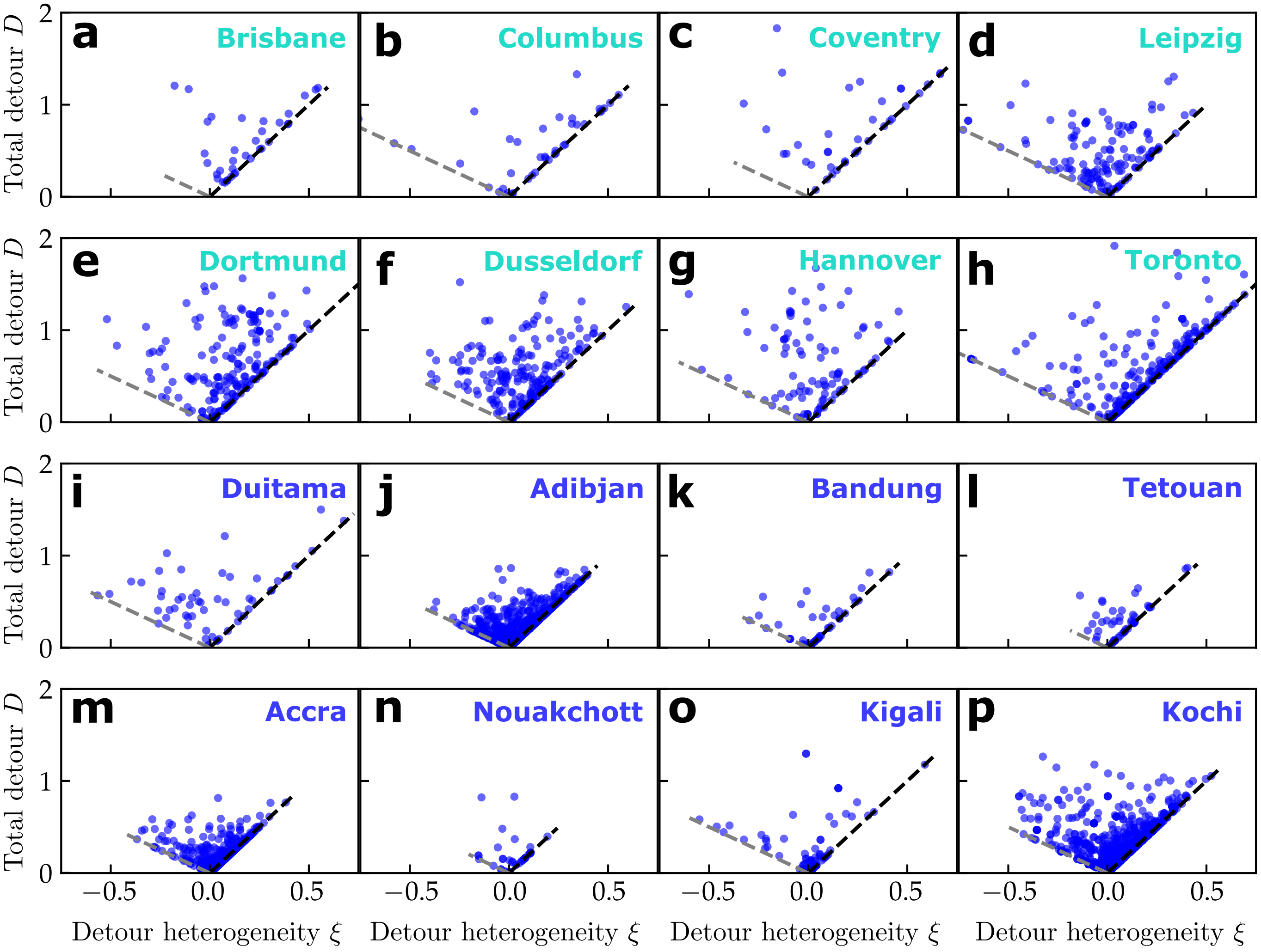

Supplementary Figure S15. Total detour D and detour heterogeneity ξ for individual routes in different cities. **a-h** Total detour and detour heterogeneity of routes in cities with formal transport. **i-p** Total detour and detour heterogeneity of routes in cities with informal transport. Across all cities, routes are mostly concentrated near the line of maximum ξ (black dashed). Cities with informal transport (panels i-p) exhibit consistently lower values of D and ξ.

The relations discussed above (compare Fig. S12) become more apparent in the corresponding contour density plots for individual routes across different cities, Fig. S16. Cities with formal routes (Fig. S16a-h) display elevated values for both D and ξ. Conversely, cities characterized by informal transport consistently exhibit lower values for both ξ and D, as evidenced by the densely concentrated contour densities for these cities (Fig. S16i-p).

The contrast between public transportation in cities of the Global South and the Global North is starkly apparent in the combined contour density plots representing routes in both regions. Routes in the Global North exhibit a wide dispersion, with many routes displaying higher values for both ξ and D whereas routes in the Global South are notably concentrated towards lower values for these variables, also visible in the marginal distributions of both quantities (Fig. S17).

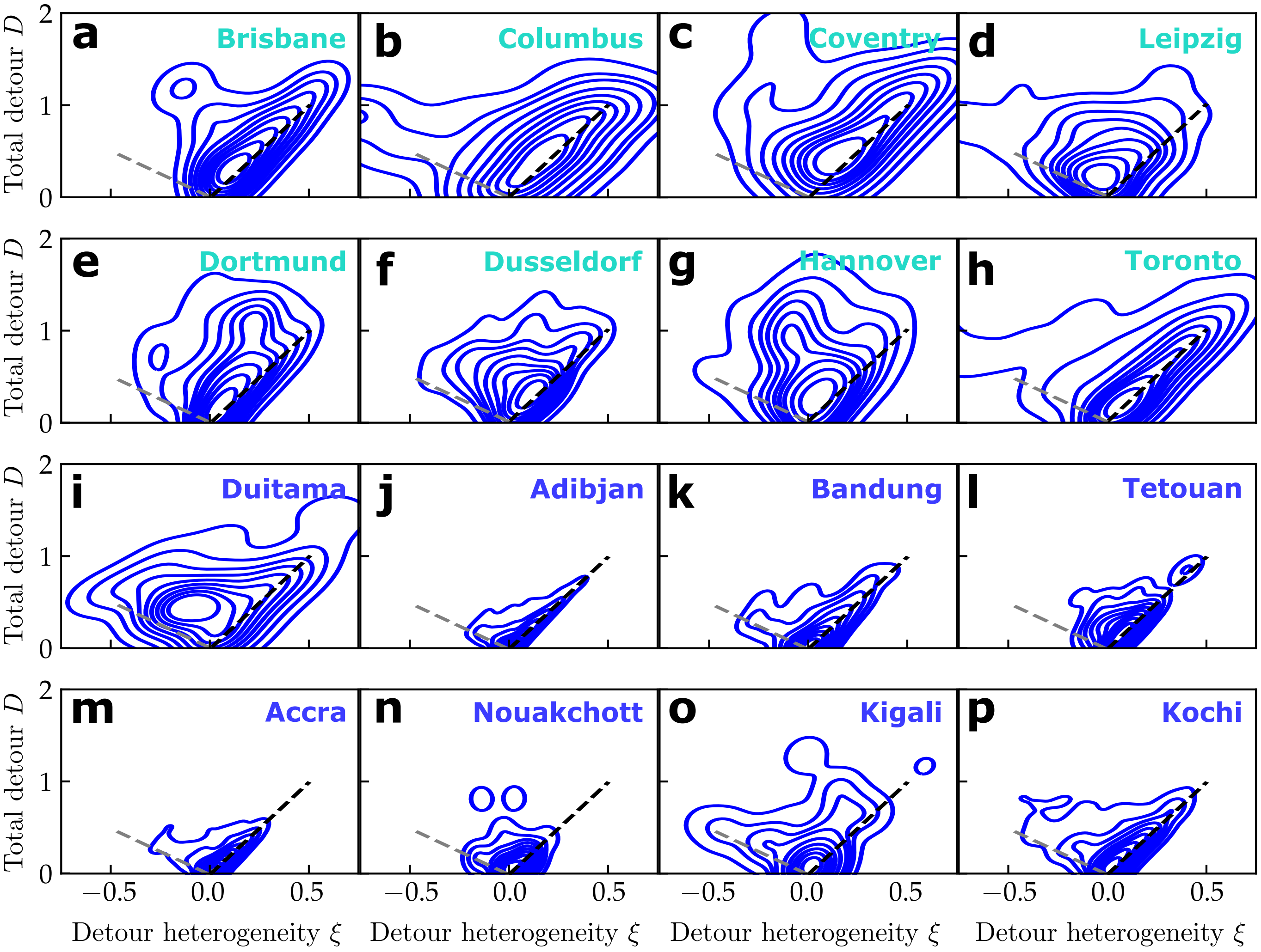

Supplementary Figure S16. Total detour D and detour heterogeneity ξ contour density for individual routes in different cities. **a-h** Total detour and detour heterogeneity of routes in cities with formal transport. **i-p** Total detour and detour heterogeneity of routes in cities with informal transport. Across all cities, routes are mostly concentrated near the line of maximum ξ (black dashed). Cities with informal transport (panels i-p) exhibit consistently lower values of D and ξ.

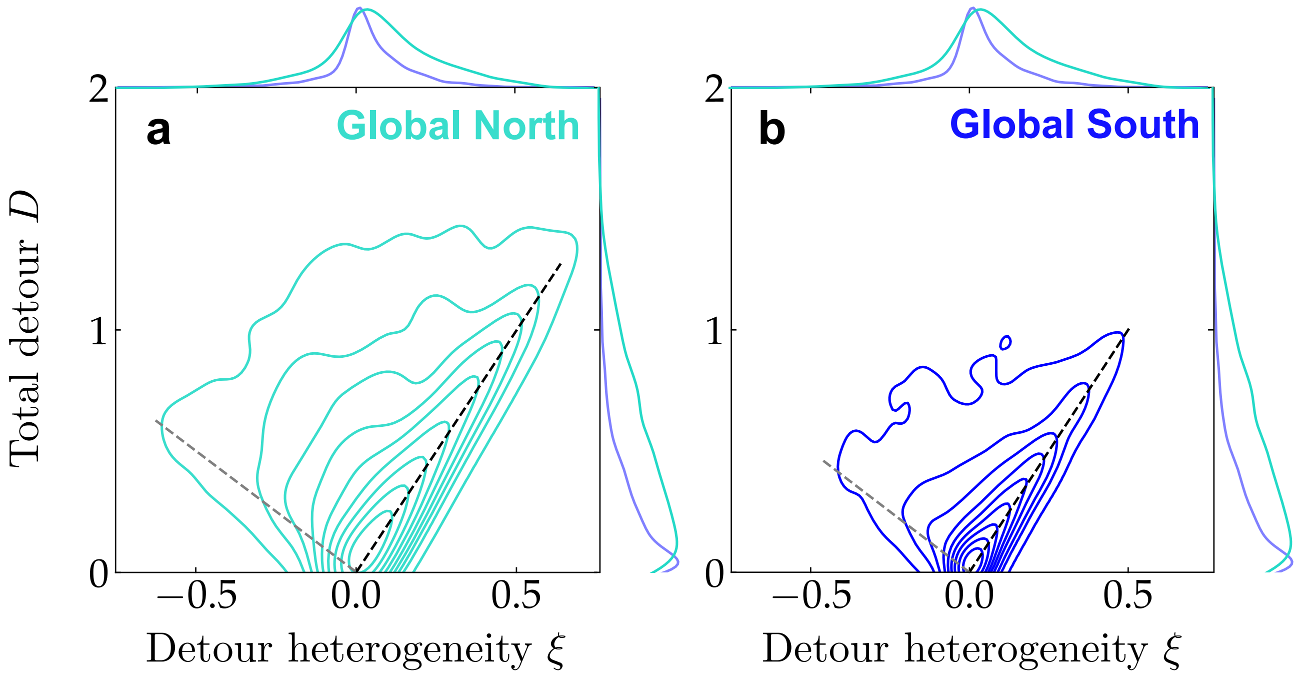

Supplementary Figure S17. Contour density plots for **a** all the formal routes in the Global North (light blue) and **b** all informal routes in the Global South (dark blue). Self-organized routes in the Global South consistently exhibit lower values for both the total detour D and detour heterogeneity ξ. Curves above and to the right of the main plot illustrate the marginal distributions of the detour heterogeneity and the total detour, respectively. The wider spread and larger total detours of cities with formal transport (Global North, light blue) compared to informal transport (Global South, dark blue) is clearly visible.

SUPPLEMENTARY NOTE 12: ROUTE INTERCONNECTIVITY

In the main manuscript we show the analysis of the structure of public transport routes in terms the detour along the route compared to direct trips. However, most of the potential trips in a city are not directly covered by a route. Achieving city-wide accessibility requires designing routes that intersect with each other, allowing passengers to easily transfer to other routes to reach their destination. We measure the intermediate routes C of the routes in a city as another structural property of the public transport routes. C quantifies the number of transfers needed to travel from any random starting bus to another within the network (compare Methods and reference [53] in the main manuscript). To compute the intermediate routes C, we compute how many routes C_{AB} are required to switch from a route A to a route B (see Methods in main manuscript). From this data, we compute the average interconnectivity $C_A = \frac{1}{N_{\text{routes}}-1}\sum_B C_{AB}$ of a route A by averaging over all other routes B.

We note that due to limited data availability, especially for informal services, we neglect the temporal aspect of connections between different services. Moreover, our analysis only takes into account routes of buses for both formal and informal transport. Especially larger cities with formal public transport often also have higher-capacity services, such as trams or light rail, that form another layer of the public transportation network. Conversely, cities with informal transport often also have smaller services with various other types of vehicles such as auto rickshaw and motorbikes that provide mobility services complementary to the routes of larger buses and enhance the interconnectivity in the city.

Figure S18 shows the resulting distribution of the intermediate routes C for all cities (compare Fig. 3 in the main manuscript). The networks in all cities are densely connected and one to two transfers are sufficient to reach almost all destinations from any starting route in most cities. In contrast to the detour heterogeneity and the total detour, arranging cities in increasing order of their median intermediate routes $\langle C\rangle$ results in a mixed order of cities with formal and informal public transport. Both types of cities have a similar aggregated median number of intermediate routes of $\langle C \mid |\text{formal}\rangle = 0.675$ and $\langle C \mid |\text{informal}\rangle = 0.670$. The Mann-Whitney U test also clearly rejects the hypothesis that informal public transport is better connected (smaller $\langle C\rangle$) than formal public transport with a p-value of 0.48. The number of intermediate routes between different bus routes is thus similar for formal and informal public transport. Significantly, despite informal transport routes mostly being operated by individual drivers or small collectives of drivers without central organization across a city, informal transport routes still self-organize to achieve comparable interconnectivity levels relative to formal transport while at the same time having fewer detours.

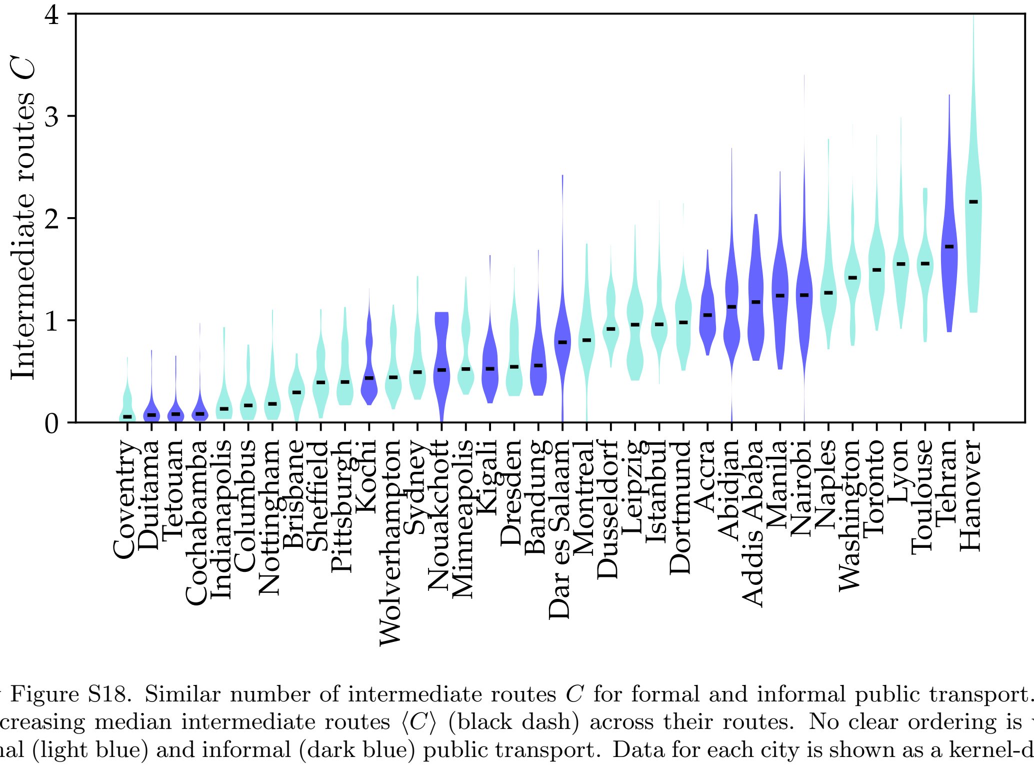

Supplementary Figure S18. Similar number of intermediate routes C for formal and informal public transport. The cities are arranged by increasing median intermediate routes $\langle C\rangle$ (black dash) across their routes. No clear ordering is visible between cities with formal (light blue) and informal (dark blue) public transport. Data for each city is shown as a kernel-density estimate with the horizontal width of the distribution proportional to the number of routes in the city with the respective value of the intermediate routes (not comparable across cities).

SUPPLEMENTARY NOTE 13: POPULATION WEIGHTED MEASURES

Routes of formal public transport services are designed to provide access to mobility to the whole population of the city. In contrast, routes of informal public transport self-organize to offer mobility services according to the demand to maximize their income. As a result, the properties of the resulting routes may differ from a purely structural perspective and when viewed with the population distribution in mind.

To further test the robustness of our results, we repeat the analysis of the structural properties of the routes in terms of detour heterogeneity, total detour, and intermediate routes with respect to the population distribution. We weigh each route with the total population served based on the 400m hexagons in the Kontur population data (compare Supplementary Note 3 and Methods in the main manuscript) calculated as the sum of the population in the hexagons that the route passes through. The population-weighted distribution of detour heterogeneity ξ, total detour D, and intermediate routes C are shown in Fig. S19 and numeric results are shown in Tab. S3 (compare Fig. 3 and Tab. 1 in the main manuscript as well as Fig. S18 above). In general, our previous results remain qualitatively robust and the results of the rank test remain the same for all three observables. Median values of the route heterogeneity and total detour increase slightly in the population weighted analysis. The median weighted number of intermediate routes of formal public transport decreases more strongly compared to informal public transport, indicating a better organization of highly connected routes for a larger part of the population of formal public transport routes.

Observable	Median Informal	Median Formal	p-value
Detour Heterogeneity ξ	0.011	0.070	7.0×10^{-4}
Weighted Detour Heterogeneity ξ	0.016	0.071	2.5×10^{-3}
Total Detour D	0.12	0.45	4.0×10^{-5}
Weighted Total Detour D	0.13	0.45	4.6×10^{-5}
Intermediate Routes C	0.670	0.675	4.8×10^{-1}
Weighted Intermediate Routes C	0.63	0.55	4.3×10^{-1}

Supplementary Table S3. Observable statistics. Column 1 indicates the observable measured. Column 2 and 3 show the median value for the given observable for informal and formal transport, respectively. Column 4 shows the p-value for the Mann-Whitney U rank statistics test for the given observable (compare Tab. 1 in the main manuscript).

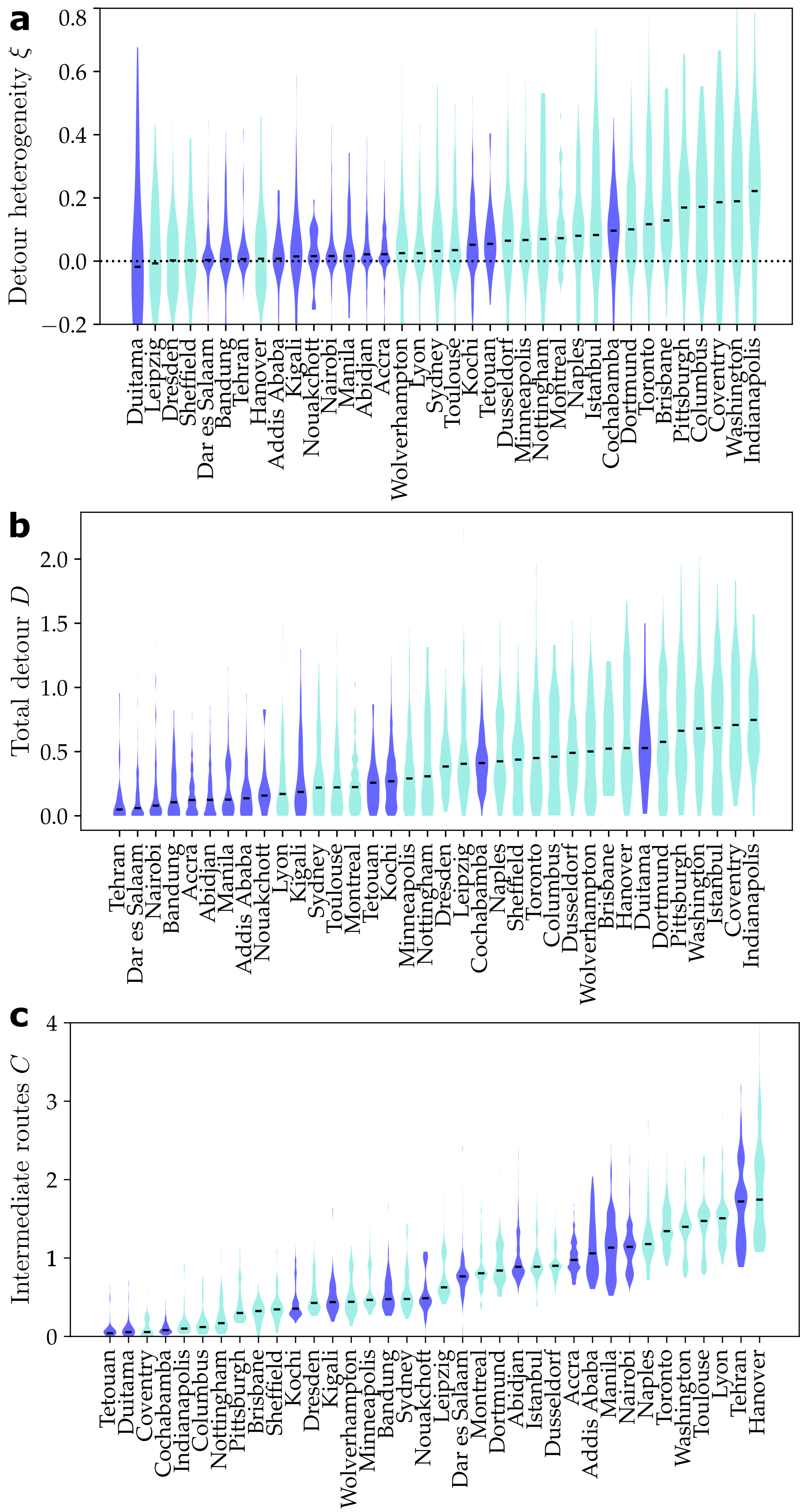

Supplementary Figure S19. Population weighted ranking of structural route properties. **a** Detour heterogeneity ξ. **b** Total detour D. **c** Intermediate routes C. The qualitative behavior of these observables is similar for both population-weighted and unweighted analysis (compare Tab. S3, Fig. 3 in the main manuscript, as well as Fig. S18). Informal transport (dark blue) consistently performs better than formal transport (light blue) in terms of lower detour heterogeneity ξ and total detour D, whereas the distribution for C is mixed across both types.
